# Post-Traumatic Epilepsy: Observations from an Urban Level 1 Trauma Center

**DOI:** 10.3390/neurolint16040063

**Published:** 2024-08-05

**Authors:** Daniel Kotas, Huaqing Zhao, John Turella, Willard S. Kasoff

**Affiliations:** 1 Department of Neurosurgery, Lewis Katz School of Medicine, Temple University, Philadelphia, PA 19140, USA; daniel.kotas@temple.edu; 2 Department of Biomedical Education and Data Science, Lewis Katz School of Medicine, Temple University, Philadelphia, PA 19140, USA; 3 Center for Biostatistics and Epidemiology, Lewis Katz School of Medicine, Temple University, Philadelphia, PA 19140, USA

**Keywords:** post-traumatic epilepsy, traumatic brain injury, follow-up

## Abstract

There are approximately 2.5 million cases of traumatic brain injury (TBI) in the U.S. each year. Post-traumatic epilepsy (PTE), a sequela of TBI, has been shown to occur in approximately 15% of TBI patients. Pre-disposing risk factors for the development of PTE include severe TBI and penetrating head injury. PTE is associated with poor functional outcomes, increased negative social factors, and mental illness. We conducted a retrospective chart review with a 5-year timeframe at an urban Level 1 Trauma Center. Patients with ICD-10-CM codes associated with TBI were identified. Patients were coded as TBI with or without PTE by the presence of codes associated with PTE. Datapoints collected included risk factors for PTE and encounters with neurologists. A total of 1886 TBI patients were identified, with 178 (9.44%) classified as TBI with PTE. The most significant risk factor associated with PTE was severe brain injury, with an odds ratio (OR) of 2.955 (95% CI [2.062,4.236]; *p* < 0.0001). Only 19 of 178 patients (10.7%) visited a neurologist beyond 6 months after TBI. Our results suggest the presence of a significant population of patients with PTE and the need for better follow-up.

## 1. Introduction

In the United States, there are approximately 2.5 million cases of traumatic brain injury (TBI) each year, with 23 million U.S. adults over the age of 40 reporting a history of TBI with loss of consciousness as of 2014 [1,2]. TBI causes significant socioeconomic burden, with 10%, 66%, and 100% of patients with mild, moderate, and severe TBI, respectively, experiencing some form of permanent disability [3,4].

Post-traumatic epilepsy (PTE), defined as the occurrence of one or more unprovoked seizures more than one-week post-TBI, is a major source of morbidity after TBI [5,6]. Estimates of PTE rates vary widely; a 2017 meta-analysis found a pooled PTE prevalence of 15% (95% CI 14–17%) among TBI patients, with a range of estimates between 1.3% and 53.3% among 20 included studies [6,7]. Although PTE accounts for less than 10% of all cases of epilepsy in the U.S., it is disproportionately responsible for preventable cases of epilepsy and cases among the young, accounting for 30% of epilepsy cases in the 15–34-year age group [6,7]

There are several well-studied predisposing factors for the development of PTE in patients with TBI. Military studies have highlighted penetrating head injury as a major risk factor, with a study of Vietnam War veterans finding that 45–53% of TBI patients in this category developed PTE [8]. Other substantial risk factors include severe TBI, intracerebral hemorrhage, skull fracture, and prior alcohol use disorder [5]. Approximately 80% of PTE cases present within the first two years post-TBI, but the risk for PTE after a severe TBI can be elevated for over 10 years [5,6,9].

PTE can be difficult to treat medically and has been associated with poor psychosocial outcomes [5,6,9]. A study using a 2010–2012 cohort from the U.S. TBI Model Systems National Database found increased rates of depression and anxiety in patients with PTE versus TBI patients without PTE [10]. Populations of patients with PTE also experience lower Satisfaction With Life Scale scores and a higher incidence of personality disorders, disinhibited behaviors, and emotional dysregulation compared with patients with TBI and no PTE [11,12,13].

Despite the negative effects of PTE, systematic efforts to identify and reach patients with, or at high risk of, PTE are lacking. We aimed to assess the existence of an untreated population of patients with PTE within the network of a large, urban Level 1 Trauma Center with the goal of providing knowledge to guide future studies and outreach. 

## 2. Materials and Methods

This study was reviewed and approved by Temple University’s IRB Committee (Protocol 29697).

The Temple University Hospital Epic database was queried from 1 January 2016 to 31 December 2020 to identify patients with TBI who subsequently developed seizures. Patients with TBI were identified by searching for ICD-10-CM diagnosis codes of S02.0, S02.1, S02.8, S02.91, S02.0XXA, S06.0–S06.9, and S06.A (see supplementary tables. Patients under 18 years old and patients with a discharge status other than alive were excluded. Patients with a previous diagnosis of epilepsy of any etiology were excluded using the diagnosis codes of R56.1 (post traumatic seizures), R56.9 (unspecified convulsions), and G40 (epilepsy and recurrent seizures). Data points were extracted from patient charts, including demographics, pre-existing conditions, risk factors, initial GCS, outpatient encounters with Neurology at least 6 months after the TBI, and the prescription of antiepileptic drugs (AEDs) at least 6 months after the diagnosis of TBI (see supplementary tables). Patients were classified as having had severe brain injury if their GCS on presentation was less than 9 [4,14,15]. Patients with TBI and the subsequent assignment of an epilepsy diagnosis code (R56.1, R56.9, or G40) within the study timeframe were considered to have developed PTE and were classified as “TBI with PTE”. All other patients were classified as “TBI without PTE”.

A separate Epic query was conducted using the same timeframe of 1 January 2016 to 31 December 2020 for patients with the specific PTE diagnosis code of R56.1. This search extracted demographics, date of diagnosis, record of any Neurology office visit, and any record of antiepileptic drug prescription.

Continuous variables were reported as the mean ± SD and categorical variables were reported as counts and percentages. Continuous variables were compared using a 2-sample student’s *t*-test, and categorical variables were compared using the Pearson chi-square test. A univariate logistic regression model was used to identify factors associated with PTE and, if significant, these variables were then tested in a multivariable analysis model. All statistical analyses were considered statistically significant if the *p* values were <0.05 without adjusting for multiple comparisons. The software used for all the analysis was SAS version 9.4 (SAS Institute Inc., Cary, NC, USA).

## 3. Results

### 3.1. Study Population

The demographic results are summarized in Table 1. A total of 1886 TBI patients were identified. The median age was 50.0 (interquartile range (IQR) 33.0, 67.0). The majority (72.7%) were male. A total of 722 patients (39.0%) were Black, 507 (27.4%) were White, and 429 (23.2%) were Latinx. The average hospital length of stay (ALOS) was 8.8 days (SD 14.6), with a median of 4.4 (IQR 2.4, 9.6).

Of the total 1886 patients, 1708 (90.6%) were classified as “TBI without PTE”. The median age of this population was 51.0 (IQR 33.0, 67.0). The majority (72.4%) were male. A total of 661 patients (39.4%) were Black, 449 (26.8%) were White, and 390 (23.3%) were Latinx. The ALOS of patients in this group was 8.0 days (SD 12.6), with a median of 4.2 (IQR 2.3, 9.0).

Of the total 1886 patients, 178 (9.4%) were identified as developing a new diagnosis of epilepsy after TBI and were classified as “TBI with PTE”. The median age of this population was 50.0 (IQR 33.0, 63.0). The majority (75.8%) were male. A total of 61 patients (34.5%) were Black, 58 (32.8%) were White, and 61 (34.5%) were Latinx. The ALOS of patients in this group was 16.1 days (SD 26.0), with a median of 7.4 (IQR 3.1, 19.6). There were no significant demographic differences between the TBI without PTE and with PTE groups as determined by comparison using 2-sample student’s *t*-testing for continuous variables and Pearson chi-square testing for categorical variables.

A total of 158 patients were identified with the diagnosis code of R56.1. Of these patients, 144 (91.1%) had AEDs in their chart and 102 (64.6%) had a record of a Neurology visit at any time within the study timeframe.

### 3.2. Risk Factors for PTE

A statistically significant association in both the univariate logistic regression and multivariable analysis models with the development of PTE was found for severe TBI. As seen in Table 2, 424 (22.5%) patients experienced severe brain injury and 83 (19.6%) developed PTE compared with 95 of 1462 (6.49%) patients without severe TBI (OR = 2.955, 95% CI [2.062, 4.236]; *p* < 0.0001) (Table 3). 

Midline shift and subdural hemorrhage were significant in the univariate but not multivariate analysis. A total of 761 patients, or 40.3% of the TBI population, experienced subdural hemorrhage. In the univariate analysis, penetrating TBI, intracerebral hemorrhage, and cerebral contusions were not found to be significantly associated with the development of PTE (Table 2), and skull fracture was found to be negatively associated with the development of PTE (OR = 0.615, 95% CI [0.435, 0.870]; *p* = 0.0059) (Table 3). No significant differences in pre-existing alcohol use disorder, stroke, or depression were found between the study groups (Table 1).

### 3.3. Neurology Follow-Up and Antiepileptic Drugs

A total of 165 out of 178 (92.7%) patients who developed PTE had at least one AED on their medication list at least 6 months after the diagnosis of TBI, compared with 1225 of 1708 patients without PTE (71.7%). Nineteen patients with PTE (10.7%) had Neurology clinic visits at least 6 months after the diagnosis of TBI compared with 89 patients without PTE (5.21%) (Table 1). Of 158 patients with the diagnosis code of R56.1, 144 (91.1%) had AEDs in their chart, and 102 (64.6%) had a record of a Neurology visit at any time within the study timeframe. Both data points were significantly associated with PTE in a multivariable logistic regression model. Neurology clinic visits at least 6 months after the diagnosis of TBI was associated with PTE, with an OR of 1.851 (95% CI [1.074, 3.188]; *p* = 0.0265). The presence of AEDs at greater than 6 months after the diagnosis of TBI was associated with PTE, with an OR of 3.817 (95% CI [2.132, 6.836]; *p* < 0.0001) (Table 3).

## 4. Discussion

Our study utilized a single hospital administrative database to examine the TBI population for rates of PTE, associated risk factors, and evidence of follow-up in a Neurology clinic after the development of PTE. Over 5 years, 178 of 1886 TBI patients were subsequently given a coded diagnosis of epilepsy, yielding an estimated rate of 9.44% (Table 1). This is consistent with previously published findings but less than the pooled prevalence of 15% reported in a meta-analysis of 20 studies [5]. It is well known that individuals of male sex are more predisposed to TBI, which was found in our study as well [7,14]. Male sex was not found to be significantly associated with the development of PTE after TBI (Table 1). Considering that TBI has been found to be most common in young adults, the median age of this study population was greater than expected, at 50.0 years (IQR 33.0, 67.0) [7]. 

There was a significant difference in length of stay (LOS) between the TBI without and with PTE groups. Patients with a presumed diagnosis of PTE had an ALOS around double that of the TBI without PTE group, at 16.1 days (SD 26.0) and 8.0 days (SD 12.6), respectively (Table 1). The difference in LOS between the TBI with and without PTE groups can likely be attributed to factors such as hospital courses complicated by early post-traumatic seizures and a higher proportion of patients with severe brain injury in the TBI with PTE group. There is variability in the LOS reported by other studies. A prospective study in Texas reported a PTE group ALOS of 20.6 days (SD 17.7) and a non-PTE group ALOS of 14.3 days (SD 11.7) [15,16]. A longitudinal study of TBI patients in Iran reported the ALOS of PTE and non-PTE groups to be 30 days (SD 16) and 24 days (SD 18), respectively [17].

The most significant risk factor associated with the diagnosis of PTE was severe brain injury, with an OR of 2.955 (95% CI [2.062, 4.236]; *p* < 0.0001) (Table 3). This is consistent with the findings of other studies, which show a pattern of severe brain injury as the risk factor most strongly associated with PTE [4,5,7,9,17]. The high proportion of patients with severe brain injury in the TBI with PTE group indicates the presence of a significant population of patients who likely experience a large burden of disease and are especially vulnerable. With both severe brain injury and PTE, this specific subgroup of patients likely experiences high rates of permanent disability and poor functional outcomes in addition to the stigma and socioeconomic ramifications of living with epilepsy [12,18,19].

One unexpected finding was that the odds of being categorized into the TBI with PTE group were found to be increased with the absence of skull fracture (OR = 1.626, 95% CI [1.150, 2.298]; *p* = 0.0059) (Table 3). Several prior studies have found skull fracture to be associated with an increased risk for the development of PTE [5,20,21,22]. In addition, the absence of statistically significant associations between intracranial hemorrhage (either intraparenchymal or subdural), midline shift or penetrating brain injury with PTE was surprising (Table 2). Considering prior data and the pathophysiology of PTE, these findings are likely spurious and due, at least in part, to the common errors of administrative databases; namely, inaccuracies in coding or loss to follow-up in this single-institution retrospective study.

Considering the lifelong challenges faced by patients living with PTE, we decided that it would be important to investigate long-term care for this group, a subject which has not been well studied. We used records of Neurology visits at least 6 months after the diagnosis of TBI as a proxy for whether patients were receiving long-term follow-up care. Only 19 of 178 patients in the TBI with PTE group (10.7%) had Neurology visits at least 6 months after the diagnosis of TBI during the approximately 5-year study timeline (Table 1). In the query conducted for patients with the specific diagnosis of post-traumatic seizures (ICD-10-CM R56.1), only 102 of 158 patients (64.6%) had at least one Neurology visit at any time during the study timeframe.

The numerous potential barriers faced by patients with PTE cannot be ignored when considering the long-term care for this group. Patients with PTE experience increased rates of depression, anxiety, emotional dysregulation, and personality disorders in addition to social stigma and limitations in employment and transportation [10,12,13,19,23]. These factors could have influenced the rates of Neurology follow-up in the TBI with PTE group by contributing to poor mental health outcomes and socioeconomic disparities. Another aspect to consider is the impact of COVID-19 pandemic stay-at-home orders that began in the U.S. in March 2020, the last year captured by this study [24]. COVID-19 had widespread impacts on the care received by patients, especially those with chronic conditions, by limiting access and imposing novel barriers to adequate healthcare [25]. 

Regardless of the underlying cause, the findings of this study indicate the presence of a general gap in the treatment of PTE, with a significant undertreated population of patients in the long term, especially, but in the short term as well. The findings of this study should encourage clinicians to focus on maintained follow-up for PTE patients. In addition, this population would benefit from further study to better characterize the reasons for the loss of follow-up, with the goal of developing targeted strategies to improve this issue. Future research could include prospective studies with better up-front capture of clinical and radiographic data as well as measures to decrease the loss of follow-up, particularly in the urban setting of a socioeconomically disadvantaged population and multiple health systems with overlapping catchment areas. This topic could also benefit from qualitative research, including patient interviews to capture individual experiences regarding potential barriers to follow-up, such as social stigma or socioeconomic challenges.

This study is subject to the typical errors of retrospective administrative database queries in capturing a clinical diagnosis (i.e., over-, under-, or mistaken coding, etc.). This could have led to an over- or underestimation of both the TBI with and without PTE groups. Studying both TBI and PTE incidence over the same 5-year period may have underestimated the PTE prevalence, since the risk for PTE after a severe brain injury can be elevated for over 10 years [5,9]. However, it is also possible that the observed epilepsy diagnoses captured early post-traumatic seizures from the index hospitalization or an additional cause of seizure, rather than true PTE, therefore overestimating the prevalence of PTE. Lastly, information was not available on follow-up by a trauma or primary care service to assess the loss to follow-up in general. 

There are also potential limitations regarding our analysis of Neurology follow-up to consider. Patients first captured in the TBI with PTE group may have received care elsewhere, resulting in the underestimation of patients with PTE receiving long-term neurological care. In addition, patients could have received care from Neurology after the study timeframe, resulting in underestimation. Patients in the TBI with PTE group captured specifically in the latter portion of the study timeframe could also have affected our analysis of Neurology follow-up, resulting in uncaptured visits and the underestimation of patients receiving long-term Neurology care. However, even if all patients captured in the last year of the study in the group of TBI with PTE with no record of Neurology visits after 6 months (41 patients) did eventually have a Neurology visit, the percentage of PTE patients with Neurology visits would increase to only 33.7%. It is also possible that the Neurology follow-up was overestimated, since information on the reason for Neurology visits was not available, and patients could have been seen for reasons other than seizure.

The difference between the query for patients with exclusively the R56.1 diagnosis code versus the main query for epilepsy in general (158 patients and 178 patients, respectively) could have been due in part to the heterogeneity in coding between physicians and specialties.

Many of these deficiencies could be addressed with a prospectively maintained database, more standardized use of coding, longer-term follow-up, and the inclusion of other regional electronic medical record databases to capture patient follow-up within other nearby health systems. These improvements, however, are extremely difficult to achieve in practice.

## 5. Conclusions

In summary, our study sought to assess for the existence of an untreated population of patients with PTE, a detrimental long-term sequela of TBI, by utilizing a single hospital administrative database to analyze the TBI population for rates of PTE, associated risk factors, and evidence of Neurology follow-up. Despite the limitations discussed above, we believe that our results strongly suggest the presence of a significant population of patients with PTE within our catchment area whose index TBI was at our urban tertiary-care Level 1 Trauma Center. Further, the limitations of our study themselves point toward the need for better systems for patient identification and follow-up to treat this population. We believe that this work can be used to guide future studies aimed at better understanding and improving the PTE treatment gap within our catchment area and similar practice settings.

## Figures and Tables

**Table 1:** Summary of demographics, pre-existing comorbidities, neurology follow-up, and AEDs for the total study population, those with TBI with PTE and those with TBI without PTE. Alcohol use disorder, stroke, and depression were conditions present prior to the TBI. Values not in parentheses include the mean, median, and raw values. Values in parentheses include the standard deviation (SD) for the means, the interquartile range (IQR) for the medians, and percentages for the raw values. The *p*-values were determined using 2-sample student’s *t*-testing for continuous variables and Pearson chi-square testing for categorical variables and are significant if <0.05.

	Total		TBI without PTE		TBI with PTE		*p*-Value
Variable	n = 1886		n = 1708		n = 178		
Age							0.18
Mean (SD)	51.3	(20.3)	51.5	(20.6)	49.6	(17.8)	
Median (IQR)	50.0	(33.0–67.0)	51.0	(33.0–67.0)	50.0	(33.0–63.0)	
Sex (%)							0.33
Male	1372	(72.7)	1237	(72.4)	135	(75.8)	
Female	514	(27.3)	471	(27.6)	43	(24.2)	
Race (%)							0.49
Black	722	(39.0)	661	(39.4)	61	(34.5)	
White	507	(27.4)	449	(26.8)	58	(32.8)	
Hispanic	429	(23.2)	390	(23.3)	61	(34.5)	
Unknown	103	(5.6)	92	(5.5)	11	(6.2)	
Other race	92	(5.0)	84	(5.0)	8	(4.5)	
LOS							<0.0001
Mean (SD)	8.8	(14.6)	8.0	(12.6)	16.1	(26.0)	
Median (IQR)	4.4	(2.4–9.6)	4.2	(2.3–9.0)	7.4	(3.1–19.6)	
Alcohol use disorder (%)							0.55
No	1854	(98.3)	1680	(98.4)	174	(97.8)	
Yes	32	(1.7)	28	(1.6)	4	(2.2)	
Stroke (%)							0.55
No	1826	(96.8)	1655	(96.9)	171	(96.1)	
Yes	60	(3.2)	53	(3.1)	7	(3.9)	
Depression (%)							0.66
No	1710	(90.7)	1547	(90.6)	163	(91.6)	
Yes	176	(9.3)	161	(9.4)	15	(8.4)	
Neurology visit > 6 months (%)							0.003
No	1778	(94.3)	1619	(94.8)	159	(89.3)	
Yes	108	(5.7)	89	(5.2)	19	(10.7)	
Antiepileptic drugs (%)							<0.0001
No	496	(26.3)	483	(28.3)	13	(7.3)	
Yes	1390	(73.7)	1225	(71.7)	165	(92.7)	

**Table 2:** Summary of injury characteristics by TBI with and without PTE. Injury characteristics reported for the total study population, those with TBI with PTE, and those with TBI without PTE. Values in parentheses are percentages of the corresponding study group. The *p*-values were determined using Pearson chi-square testing and are significant if <0.05.

	Total		TBI without PTE		TBI with PTE		*p*-Value
Variable	n = 1886		n = 1708		n = 178		
Severe brain injury (%)							<0.0001
No	1462	(77.5)	1367	(80.0)	95	(53.4)	
Yes	424	(22.5)	341	(20.0)	83	(46.6)	
Skull fracture (%)							
No	1063	(56.4)	948	(55.5)	115	(64.6)	0.020
Yes	823	(43.6)	760	(44.5)	63	(35.4)	
Midline shift (%)							0.0001
No	1815	(96.2)	1653	(96.8)	162	(91.0)	
Yes	71	(3.8)	55	(3.2)	16	(9.0)	
Brain contusion (%)							0.34
No	1842	(97.7)	1670	(97.8)	172	(96.6)	
Yes	44	(2.3)	38	(2.2)	6	(3.4)	
Penetrating injury (%)							0.33
No	1782	(94.5)	1611	(94.3)	171	(96.1)	
Yes	104	(5.5)	97	(5.7)	7	(3.9)	
Subdural hemorrhage (%)							0.0007
No	1125	(59.7)	1040	(60.9)	85	(47.8)	
Yes	761	(40.3)	668	(39.1)	93	(52.2)	
Intracerebral hemorrhage (%)							0.83
No	1839	(97.5)	1665	(97.5)	174	(97.8)	
Yes	47	(2.5)	43	(2.5)	4	(2.2)	

**Table 3:** Summary of results of a multivariable logistic regression model of PTE, including variables considered to have a statistically significant association with the development of PTE.

Variable	Odds Ratio	95% Confidence Limits		*p*-Value
Severe brain injury (Y vs. N)	2.955	2.062	4.236	<0.0001
AEDs at >6 months (Y vs. N)	3.817	2.132	6.836	<0.0001
Neurology visit at >6 months (Y vs. N)	1.851	1.074	3.188	0.0265
LOS	1.011	1.003	1.019	0.0083
Skull fracture (Y vs. N)	0.615	0.435	0.870	0.0059

## Data Availability

The data that support the findings of this study are not publicly available due to their containing information that could compromise the privacy of research participants but are available from the corresponding author (WK) upon reasonable request.

## Supplementary Materials

The following supporting information can be downloaded at: https://www.mdpi.com/article/10.3390/neurolint16040063/s1, Table S1: Query criteria for new epilepsy diagnosis after TBI, Table S2: Query criteria for epilepsy diagnosis after TBI with diagnosis code ICD-CM-R56.1, and Table S3: List of antiepileptic drugs utilized in all queries.
