# Supplementary material for: Post-Traumatic Epilepsy: Observations from an Urban Level 1 Trauma Center
Source: Neurol Int. 2024 Aug 5;16(4):845–52. doi: 10.3390/neurolint16040063 (PMC11357076; doi:10.3390/neurolint16040063)
Supplement: Supplementary file 1 [file neurolint-16-00063-s001.zip › neurolint-3075142-supplementary.pdf]

**Table S1**: General Query for New Epilepsy Diagnosis After TBI
Inclusion/Exclusion criteria:
Admission date: 1/1/2016 to 12/31/2020
Age ≥ 18
Diagnosis of TBI: S02.0 (fracture (Fx) of vault of skull), S02.1 (Fx of base of skull), S02.8 (Fx of other specified skull and facial bones), S02.91 (Unspecified Fx of skull), S02.0XXA (Fx of vault of skull, initial encounter for closed fracture), S06.0 – S06.9 (intracranial injury), or S06.A (traumatic brain compression and herniation)
Exclude patients expired on admission
Exclude patients with previous diagnosis of: R56.1, R56.9, or G40
Report:
Demographics:
MRN
Zip code of residence
Race
Ethnicity
Gender
Age
Date of birth
Pre-existing conditions:
Alcohol use disorder
Previous stroke
Depression
Previous history of TBI
Risk Factors:
Skull fracture: S02 (Fracture of skull and facial bones)
Midline shift: S06.1 (Traumatic cerebral edema), S06.A1 (Traumatic brain compression with herniation)
Brain contusion: S06.31 – S06.339, S06.37 – S06.389
S06.31 (Contusion and laceration of right cerebrum), S06.32 (Contusion and laceration of left cerebrum), S06.33 (Contusion and laceration of cerebrum, unspecified)
Penetrating injury: X93.XXXA, X95.9XXA, X99.0XXA, X99.1XXA, X99.8XXA, X99.9XXA, Y24.9XXA, Y35.093A
X93.XXXA (Assault by handgun discharge), X95.9XXA (Assault by unspecified firearm discharge, initial encounter), X99.0XXA (Assault by sharp glass), X99.1XXA (Assault by knife), X99.8XXA (Assault by other sharp object), X99.9XXA (Assault by unspecified sharp object), Y24.9XXA (Unspecified firearm discharge, undetermined intent), Y35.093A (Legal intervention involving other firearm discharge, suspect injured)
Subdural hemorrhage: S06.5 (traumatic subdural hemorrhage)
Intracerebral hemorrhage: S06.36 (traumatic hemorrhage of cerebrum)
Severe brain injury (GCS < = 8)
Injury/Disease course:
PTE: R56.1, R56.9, or G40 AFTER TBI diagnosis
Y/N data point for each code

GCS on admission and last reported
Craniotomy for trauma:
61312, 61313, 61314, 61315, 61322, 61323
Y/N for each code
Encounters or appointment with neurology
At least 6 months after TBI
Antiepileptic drugs prescribed prior to admission and at discharge (see list)
Y/N any time

**Table S2**: Query for R56.1 Epilepsy Diagnosis After TBI<br>ICD-CM-R56.1
Inclusion/Exclusion criteria:
R56.1
Admission date: 1/1/2016 to 12/31/2020
Report:
Demographics
Date of diagnosis
Any neurology visits?
Date of last neurology visit
Antiepileptic drugs
Y/N any time

**Table S3:** Antiepileptic Drugs
BRIVARACETAM
CARBAMAZEPINE
CENOBAMATE
CLOBAZAM
CLONAZEPAM
DIAZEPAM
DIVALPROEX SODIUM
ESLICARBAZEPINE ACETATE
FELBAMATE
LACOSAMIDE
LAMOTRIGINE
LEVETIRACETAM
OXCARBAZEPINE
PERAMPANEL
PHENYTOIN
PHENYTOIN SODIUM
PHENYTOIN SODIUM EXTENDED
TOPIRAMATE
VALPROIC ACID
VALPROIC ACID (AS SODIUM SALT)

VIGABATRIN<br>ZONISAMIDE
